# Upregulated lncRNA LINC01128 in colorectal cancer accelerates cell growth and predicts malignant prognosis through sponging miR-363-3p

**DOI:** 10.1007/s00432-024-05804-4

**Published:** 2024-05-26

**Authors:** Xiaohu Zhou, Yanhui Li, Lei Wu, Chunyan Tian, Xiaoliang Wu

**Affiliations:** 1 Department of General Surgery, The Affiliated Xuzhou Municipal Hospital of Xuzhou Medical University, Xuzhou First People’s Hospital, No. 269, University Road, Xuzhou, 221000 Jiangsu China; 2 Department of Pathology, Shijie Hospital of Dongguan City, Dongguan, Guangdong China; 3 Department of Acupuncture and Rehabilitation, Affiliated Hospital of Nanjing University of Chinese Medicine, No. 155, Hanzhong Road, Nanjing, 210029 Jiangsu China

**Keywords:** Colorectal cancer, lncRNA LINC01128, miR-363-3p, Prognosis, Migration, Invasion

## Abstract

**Purpose:**

Colorectal cancer (CRC) refers to high-mortality tumors arising in the colon or rectum with a high rate of recurrence. The involvement of long non-coding RNAs (lncRNAs) contributes to the treatment and prognosis evaluation of CRC, and brings a new direction for the radical cure of patients. To identify the pathological mechanism and regulation of lncRNA LINC01128 (LINC01128) on CRC cells, and analyze its potential prognostic value.

**Methods:**

LINC01128 level in tissue and cell specimens from 122 CRC patients was evaluated by RT-qPCR. The clinical significance and prognostic value of LINC01128 in CRC were analyzed via Kaplan–Meier and Cox analysis. CCK8 and Transwell assays were used to study the function of LINC01128 in vitro. The relationship between LINC01128 and miR-363-3p was confirmed by luciferase reporter gene assay.

**Results:**

The overexpression of LINC01128 is associated with TNM stage and lymph node metastasis in CRC patients. Silencing LINC01128 inhibited the proliferation and metastasis of CRC cells. In addition, LINC01128 directly targeted and negatively regulated the miR-363-3p expression, while miR-363-3p inhibitor restored the inhibitory function of LINC01128.

**Conclusion:**

As an independent prognostic factor of CRC, upregulation of LINC01128 predicts poor prognosis and accelerates tumor deterioration through miR-363-3p.

**Supplementary Information:**

The online version contains supplementary material available at 10.1007/s00432-024-05804-4.

## Introduction

Colorectal cancer (CRC) is a dangerous disease of the digestive tract, and the number of new cases and deaths is increasing gradually (Zheng et al. 2021). CRC is the second leading cause of cancer deaths in the United States, and more than 34% of CRC patients died in 2023, according to a new report (Siegel et al. 2023). The new incidence of CRC in China was 12.2% by 2020, and the incidence in urban population was much higher than that in rural areas (Wang et al. 2023b; Zhang et al. 2022). Currently, it is believed that family heredity, dietary habits, lifestyle and environment may be the predisposing factors of CRC, but the specific etiology has not been clarified (Zhou et al. 2022b). Despite the high cure rate of CRC patients treated in the early stage, the 5-year survival rate of most patients with advanced or metastatic disease is only 10% (Zhao et al. 2022). Based on this, the identification of patients at risk of recurrence by sensitive biomarkers has key practical implications for the treatment of CRC.

Long non-coding RNA (LncRNA) is one of the hot topics in the field of tumor molecular mechanism research in the past decade, which has been confirmed to be closely related to the occurrence and development of a variety of tumors (Zhu et al. 2019). There has been evidence that the dysregulation of lncRNA expression may stimulate the proliferation and promote the growth of CRC cells. For example, lncRNA LINC00114, TINCR and MIR4435-2HG were all confirmed to affect the biological functions of CRC cells and may be candidates for the diagnosis and prognosis of CRC (Liu et al. 2020; Shen et al. 2020; Zhang et al. 2019). In addition, the functions of circularRNAs (circRNAs) in CRC have also been introduced. For example, Chen et al. confirmed the potential of elevated expression of circNCOA3 in CRC drug resistance, patient prognosis and treatment in the latest report (Chen et al. 2024). LncRNA LINC01128 (LINC01128) was believed to be associated with human diseases, which was abnormally expressed in a variety of tumors (Li et al. 2020; Rout et al. 2022; Yan et al. 2021). Moreover, LINC01128 was mentioned as a possible association with cancer staging (Zhou et al. 2020). Meanwhile, lncRNAs play the role of microRNA (miRNA) sponge and have an impact on the process of diseases. For example, LINC01128 upregulated SFN expression by binding to miR-383-5, which has the ability to promote the carcinogenesis of cervical cancer (Hu et al. 2019). Abnormal levels of LINC01128 are likely involved in the progression and prognosis of CRC, but there is currently no direct evidence for this.

Therefore, the present study examined the LINC01128 level in the collected specimens and focused on the function of its abnormal expression in CRC. By further elaborating the relationship and regulatory mechanism between LINC01128 and its downstream miRNA (miR-363-3p), this study provides a reference for exploring the prognostic and therapeutic factors in CRC.

## Materials and methods

### CRC sample collection and follow-up

Tissue samples (both normal and tumor tissues) were collected from CRC patients undergoing surgical treatment at The Affiliated Xuzhou Municipal Hospital of Xuzhou Medical University (March 2016–November 2018). All tissue samples obtained during surgery were rapidly stored in liquid nitrogen for use. To ensure the accuracy of the study, the 122 CRC patients included should ensure that: CRC patients were diagnosed; All patients had not received surgical treatment before the study; All patients had no other major illnesses. LINC01128 expression and the CRC patients’ clinical characteristics data are recorded in Table 1.

**Table 1:** Association of LINC01128 with patients’ clinicopathological features

	Case (n = 122)	LINC01128 expression		*P*
		Low (n = 58)	High (n = 64)	
*Age*				0.835
≤ 60	64	31	33	
> 60	58	27	31	
*Sex*				0.676
Male	67	33	34	
Female	55	25	30	
*Tumor size*				0.125
≤ 5	69	37	32	
> 5	53	21	32	
*Tumor location*				0.457
Colon left	42	23	19	
Colon right	39	16	23	
Rectum	41	19	22	
*Differentiation*				0.131
Well, moderate	67	36	31	
Poor	55	22	33	
*TNM stage*				0.024
I–II	87	47	40	
III–IV	35	11	24	
*Lymph node metastasis*				0.017
Absent	91	49	42	
Present	31	9	22	

In order to timely understand the prognosis of the CRC patients, we kept communication with the patients for 5 consecutive years after the operation and collected clinical data.

### Cell culture

Colonic epithelial cell NCM460 and colorectal cancer cell SW620, T84, SNU-503, and SW1463 were all provided by ATCC. The above cells were incubated in DMEM medium containing 10% FBS, and the incubator temperature was set at 37 °C.

### Real-time qPCR assay

Total RNA in the specimens was extracted by TRIzol Reagent (Invitrogen, USA) and then reverse transcribed into cDNA using PrimeScript™ RT Reagent Kit (Takara, Japan). The RT-qPCR system was configured using SYBR Premix Ex Taq™ II Kit (Takara, Japan), and the amplification reactions were performed on a real-time qPCR instrument. The contents of LINC01128 and miR-363-3p were calculated by the 2^−ΔΔCt^ method using GAPDH or U6 as an internal reference.

### Cell transfection

CRC cells cultured to logarithmic phase were seeded into 6-well plates and transfected with control, negative control (si-NC), or silenced LINC01128 (si-LINC01128) using lipofectamine 2000 (Invitrogen, USA). And si-NC and si-LINC01128 were provided by Ribobio company (Guangzhou, China). The corresponding transfected cells were obtained after 48 h.

### Cell proliferation assay

DMEM medium (including FBS) was added to 96-well plates, and then CRC cells were cultured at 37 °C with 5% CO_2_. Next, CRC cells were treated with CCK8 solution, and the absorbance at 450 nm was measured.

### Cell transwell assay

DMEM medium and CRC cells were placed in the upper part of the Transwell chamber (Corning, USA), and FBS and DMEM medium were filled into the lower part. After 2–3 days of incubation at the appropriate temperature (37 °C), the cells were stained with 0.1% crystal violet and counted under a microscope. The level of invasion of CRC cells was examined after addition of Matrigel to the upper layer of the Transwell.

### Dual-luciferase reporter assay

The binding sites of LINC01128 and miR-363-3p were amplified and transferred into the pmirGLO plasmid to obtain the wild-type LINC01128 vector (WT-LINC01128), and LINC01128 mutant vector (MUT-LINC01128) was constructed by cloning the binding site of LINC01128. The above vectors were co-transfected with miR-363-3p mimic/inhibitor into T84 cells with the involvement of transfection reagent lipofectamine 2000. Luciferase activity was measured by a fluorescence detector (Promega, USA).

### Statistical analysis

The experimental results were processed and plotted by SPSS 10.0 and GraphPad 20.0 software. Chi-square test, Kaplan–Meier curve and Cox regression analysis were used to evaluate the clinical data of CRC patients. Differences between groups were analyzed using Student's t-test or one-way ANOVA, and each group of experiments was repeated more than three times. The downstream targets of LINC01128 were analyzed using the online databases (ENCORI and DIANA Tools). *P* < 0.05 was considered statistically significant.

## Results

### Expression of LINC01128 and miR-363-3p in CRC tissues

**Fig. 1:**
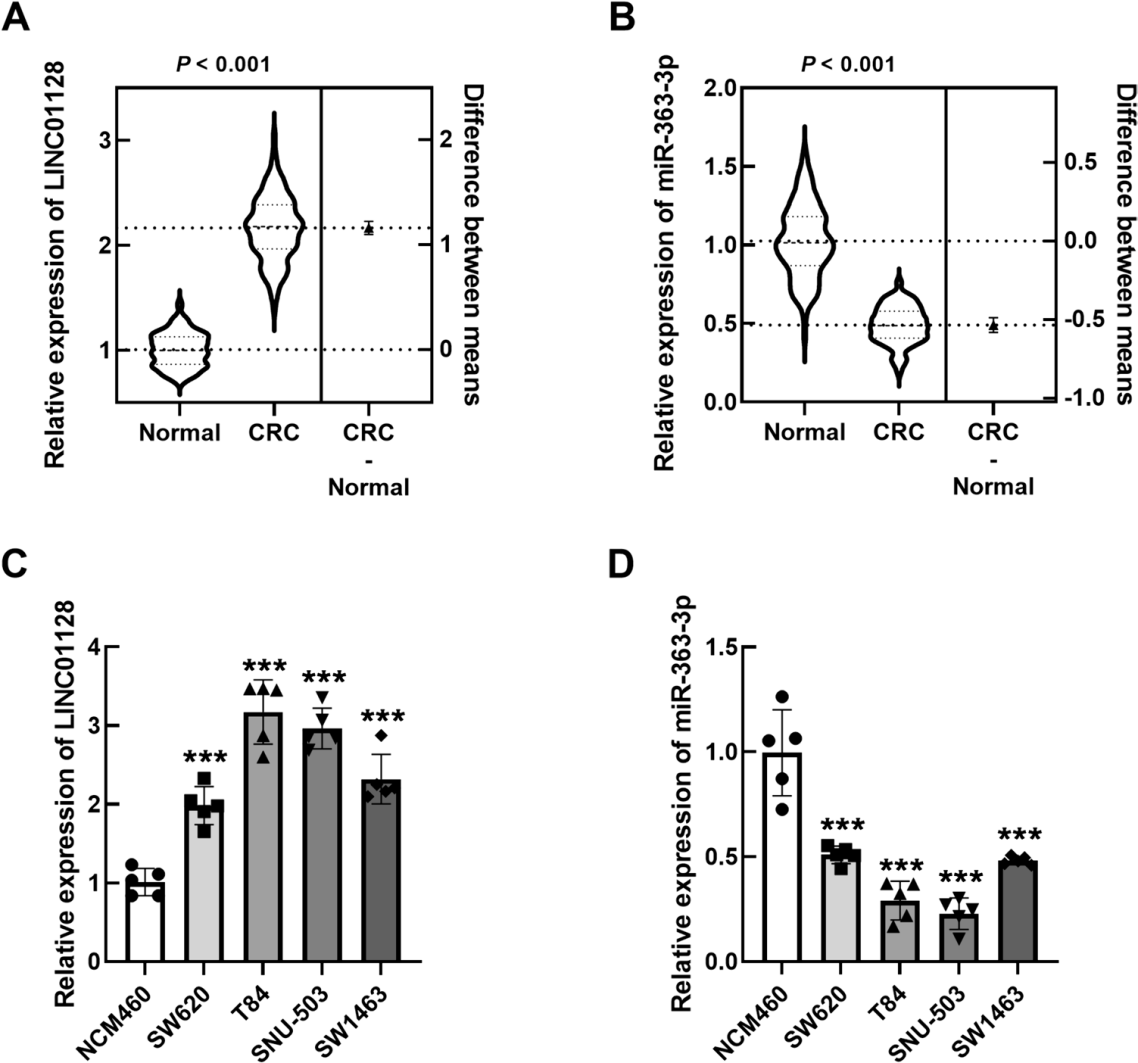
Levels of LINC01128 and miR-363-3p in CRC samples. **A, B** LINC01128 and miR-363-3p in normal and CRC tissues. **C, D** LINC01128 and miR-363-3p in normal and CRC cells. ***P < 0.001

LINC01128 was prominently upregulated in CRC tissues compared to normal tissues (Fig. 1A), whereas miR-363-3p was significantly reduced (Fig. 1B). Similarly, the expression trend was similar in CRC cells (Fig. 1C, D).

### Prognostic potential of LINC01128 in CRC

According to the mean expression of LINC01128 in CRC tissues, patients were divided into low-LINC01128 group and high-LINC01128 group. Among the clinicopathological features of CRC patients, abnormal LINC01128 level was strongly correlated with TNM stage (*P* = 0.024) and lymph node metastasis (*P* = 0.017) in Table 1.

**Fig. 2:**
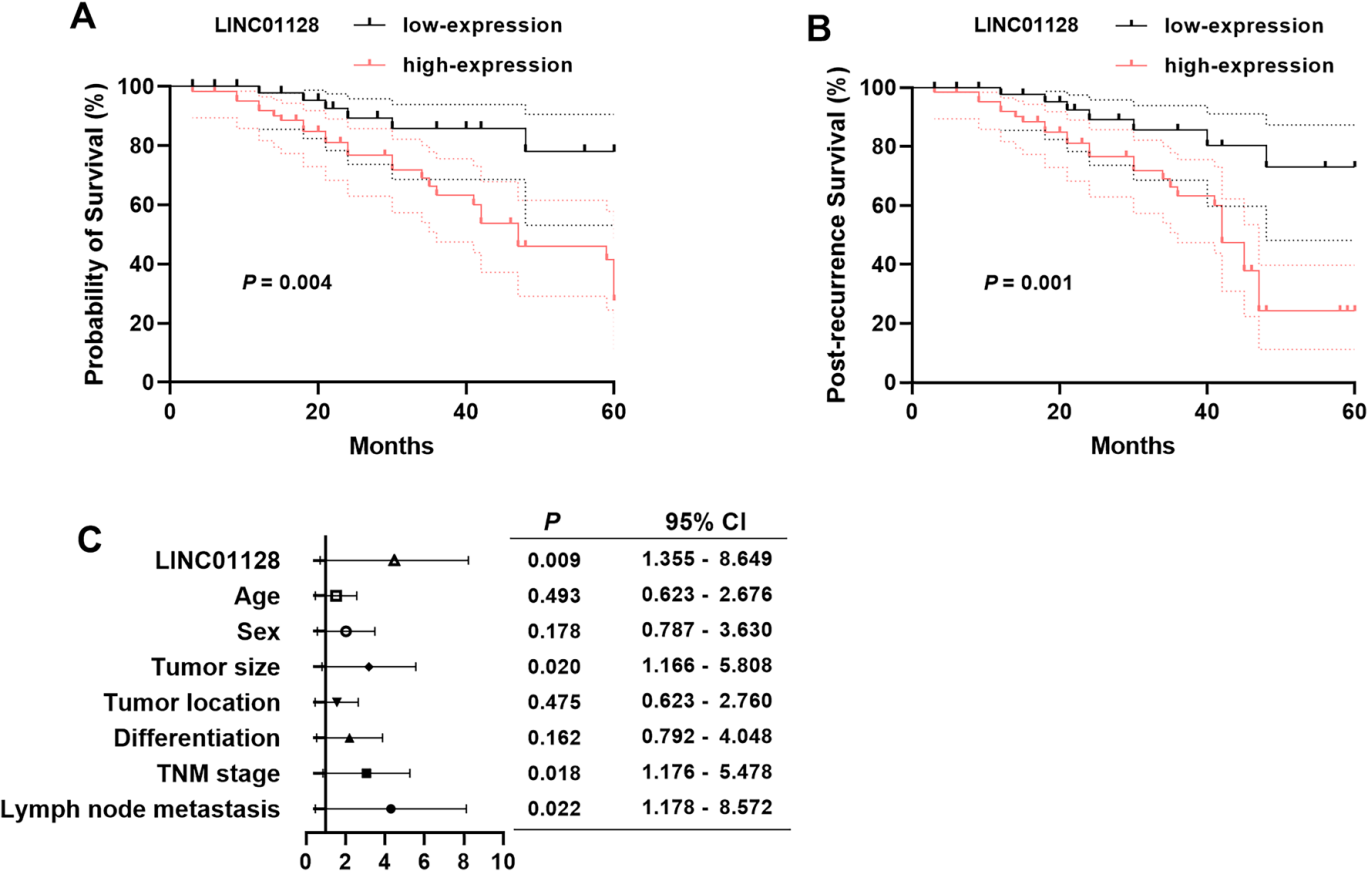
The prognostic value of LINC01128 in CRC patients. **A** Kaplan–Meier curve of LINC01128 with low and high expression (*P* = 0.004). **B** Probability of survival after recurrence in CRC patients (*P* = 0.001). **C** Forest plot of clinical indicators in CRC patients

According to the Kaplan–Meier curve drawn from the follow-up survey results, high LINC01128 expression indicated poor prognosis (*P* = 0.004, Fig. 2A). Meanwhile, statistical analysis of the post-recurrence survival rate revealed that the survival rate after recurrence was significantly decreased in the high LINC01128 expression group (*P* = 0.001, Fig. 2B). Furthermore, LINC01128 (*P* = 0.009) was found to be an independent prognostic factor for CRC with tumor size (*P* = 0.020), TNM stage (*P* = 0.018) and lymph node metastasis (*P* = 0.022) by multivariate COX analysis (Fig. 2C, Table 2).

**Table 2:** Multivariate Cox analysis of clinical characteristics in relation to overall survival

Items	Multivariate Cox analysis		
	HR	95% CI	*P*
LINC01128	3.424	1.355–8.649	0.009
Age	1.291	0.623–2.676	0.493
Sex	1.690	0.787–3.630	0.178
Tumor size	2.602	1.166–5.808	0.020
Tumor location	1.311	0.623–2.760	0.475
Differentiation	1.790	0.792–4.048	0.162
TNM stage	2.538	1.176–5.478	0.018
Lymph node metastasis	3.177	1.178–8.572	0.022

### Regulation of silencing LINC01128 in CRC cells

**Fig. 3:**
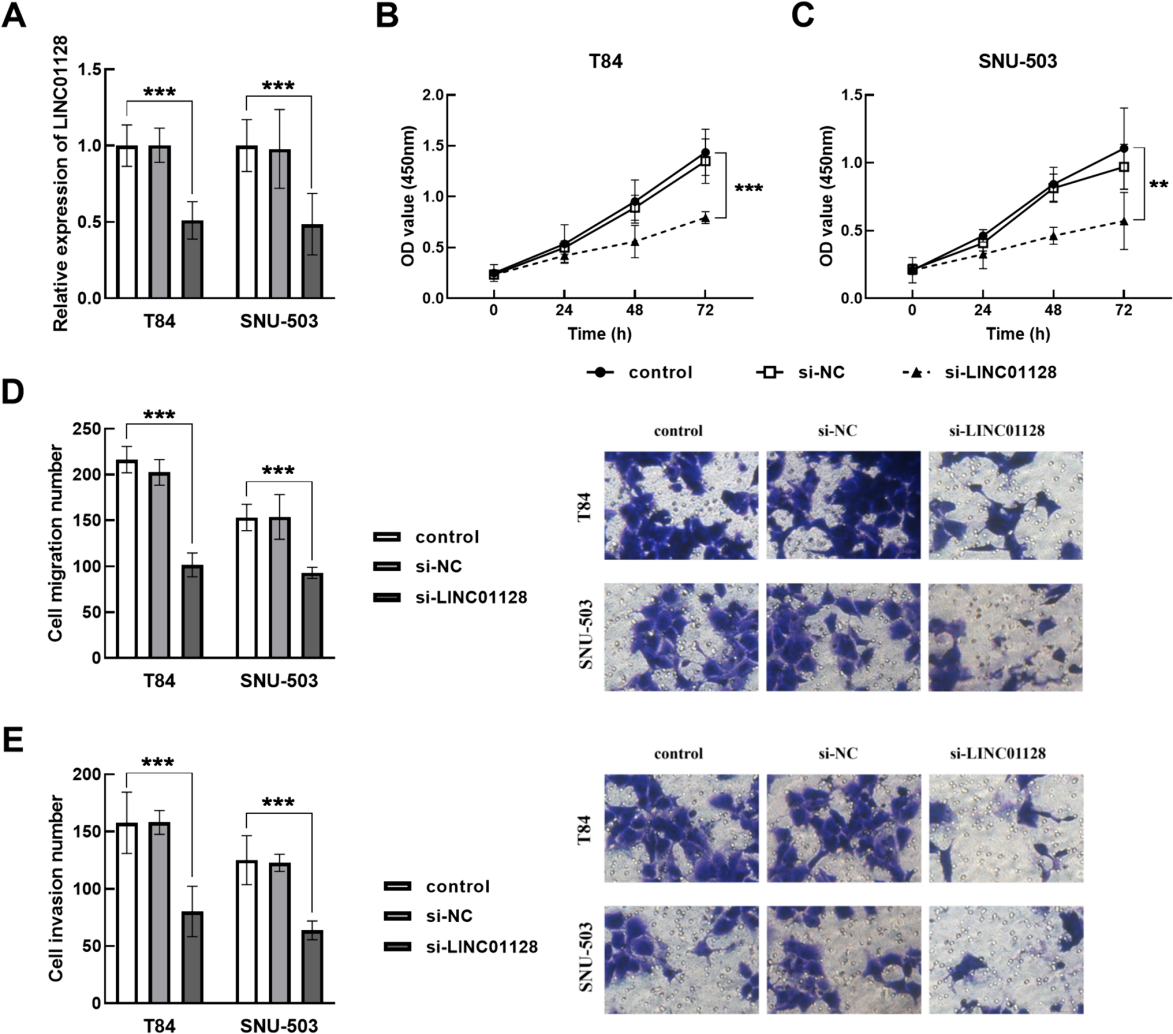
Effect of LINC01128 knockdown on cell metastatic viability. **A** Transfection results of silenced LINC01128 in T84 and SNU-503 cells. **B, C** Cell proliferation was detected by CCK8 assay. Downregulation of LINC01128 suppressed the migration (**D**) and invasion (**E**) levels of T84 and SNU-503 cells. **P < 0.01, ***P < 0.001

After transfection of si-LINC01128 into T84 and SNU-503 cells, the results were shown in Fig. 3A. Knockdown of LINC01128 notably inhibited the proliferation of T84 (Fig. 3B) and SNU-503 cells (Fig. 3C). In addition, the migration level of T84 and SNU-503 cells in the si-LINC01128 group was significantly reduced, and the invasion level of cells was also significantly inhibited after transfection of silencing LINC01128 (Fig. 3D, E).

### Targeting relationship between LINC01128 and miR-363-3p

Venn diagram showed that there may be five downstream targets of LINC01128, as predicted by ENCORI and DIANA Tools websites (Fig. 1A Supplementary). Furthermore, RT-qPCR method elucidated that miR-25-3p and miR-32-5p levels were similar in normal and CRC groups (Figs. 1B-1C Supplementary). miR-92a-3p and miR-92b-3p levels were relatively enhanced in CRC, while miR-363-3p expression was significantly downregulated in CRC (Figs. 1D–1F Supplementary).

**Fig. 4:**
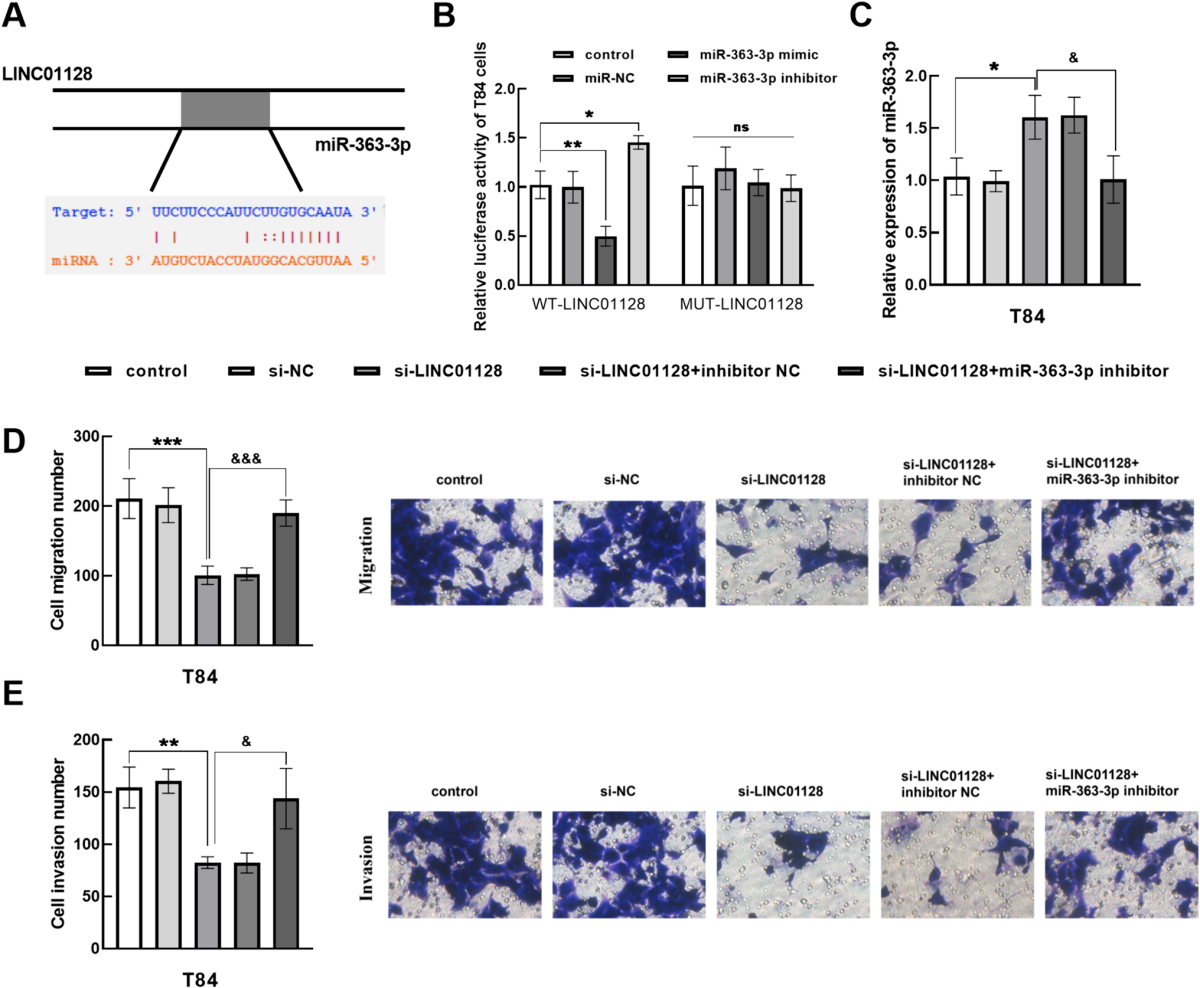
Regulatory effect of LINC01128 on miR-363-3p. **A** There are binding sites between LINC01128 and miR-363-3p. **B** Determination of luciferase activity in WT-LINC01128 and MUT-LINC01128. C. Relative expression of miR-363-3p in T84 cells. D-E. Transfection of miR-363-3p inhibitor restored the inhibition of CRC cells by si-LINC01128. ^ns^*P* > 0.05, *P < 0.05, **P < 0.01, ***P < 0.001. ^&^*P* < 0.05, ^&&&^*P* < 0.001

Through bioinformatics online prediction, LINC01128 and miR-363-3p have binding sites (Fig. 4A). Figure 4B confirmed that the luciferase activity of WT-LINC01128 was inhibited by miR-363-3p overexpression but increased by miR-363-3p silencing. Besides, after transfection of si-LINC01128, the level of miR-363-3p in the cells was increased, while transfection with miR-363-3p inhibitor, the level of miR-363-3p in T84 cells decreased (Fig. 4C). Silencing LINC01128 significantly inhibited the migration ability of T84 cells, which was reversed by transfection of miR-363-3p inhibitor in Fig. 4D. Similarly, low expression of miR-363-3p also restored the suppression of T84 cell invasion by si-LINC01128 in Fig. 4E.

## Discussion

In the treatment of patients with CRC, comprehensive treatment based on surgery is the main means at present(Wang et al. 2023a). However, the recurrence rate of postoperative CRC patients is high, especially in the middle and advanced stages, and the prognosis is not ideal. In order to timely understand the situation of patients, so as to control the recurrence and metastasis rate of tumors, people have explored the molecular mechanism of different cancers through the application of lncRNAs. For example, lncRNA KCNQ1OT1 was proposed as a diagnostic and prognostic factor in colon adenocarcinoma and rectal cancer, which played a role in enhancing drug resistance in tumor cells (He et al. 2022). lncRNA LOC441461 was revealed to regulate and promote the proliferation and motility of CRC cells (Wang et al. 2020). LINC01128 is a novel lncRNA located in human chromosome 1p36.33 (Zhong et al. 2022). LINC01128 was found to affect the activity of human chorionic trophoblast cells via targeting miR-16 (Zhao et al. 2021). In addition, LINC01128 was reported in pancreatic adenocarcinoma, breast cancer, and diabetes (Deng et al. 2022; Rout et al. 2022; Wang et al. 2021). This study showed that LINC01128 was overexpressed in CRC, and this upregulation trend was also closely related to some of the clinicopathological characteristics of the included patients, such as TNM stage and lymph node metastasis. Futhermore, the 5-year survival of CRC patients is shorter when LINC01128 expression was elevated, suggesting that upregulation of LINC01128 was detrimental to patients' treatment and survival after recurrence. Similarly, the worsening prognosis of pancreatic cancer patients has been linked to the outstanding expressed LINC01128 as mentioned by Zhong et al. (2022).

The dissemination of tumor cells is often the most dangerous process in tumor development (Zheng et al. 2023). Once tumor cells invade and metastasize, the patient's body may cause different degrees of damage. In the investigation of the pathological mechanism of CRC cells, silencing lncRNA MIR155HG was thought to negatively regulate the growth and metastasis of CRC cells via the miR-650/ANXA2 axis (Zhou et al. 2022a). Downregulation of FTX has an inhibitory effect on the viability and metastasis of CRC cells, which may slow the tumor progression (Chen et al. 2021). Our experiments also believed that LINC01128 knockdown markedly suppressed the biological function of CRC cells, suggesting that LINC01128 may act as a tumor promoter in CRC. Meanwhile, this study also revealed a significant downward trend of miR-363-3p in CRC tissues and cells by RT-qPCR. In a recent report by Wang et al., miR-363-3p was proposed to be decreased in colorectal cancer and may mediate tumorigenesis by directly targeting IFITM1 (Wang et al. 2023a). In addition, miR-363-3p was confirmed to be downregulated in polycystic ovary syndrome, ovarian serous cystadenocarcinoma, and head and neck squamous cell carcinoma (Li et al. 2023; Sang et al. 2023; Zhang et al. 2023). Furthermore, binding sites were predicted between miR-363-3p and LINC01128, and miR-363-3p was the downstream action target of LINC01128. LINC01128 negatively regulated the expression of miR-363-3p, while silencing miR-363-3p restored the inhibitory effect of LINC01128 knockdown on the migration and invasion of CRC cells. Thus, it is speculated that miR-363-3p is involved in the function of LINC01128 in CRC progression.

However, the number of samples included in this study is limited, and the research is mainly in vitro cell experiments, and relevant animal models need to be designed for verification. In addition, lncRNAs have also been found to have potential capabilities in the treatment of CRC drug resistance. For example, pep-AP encoded by lncRNA may enhance the sensitivity of CRC cells to oxaliplatin by regulating the pentose phosphate pathway (Wang et al. 2022). The application of lncRNA-miRNA-mRNA (LINC01128-miR-363-3p-mRNA) network in CRC is also a research hotspot in the future, which can help us to understand the molecular mechanism of tumors more systematically and provide more theoretical basis for the treatment of patients.

Overall, the available results clarified that LINC01128 was elevated, while miR-363-3p was downregulated in CRC. Knockdown of LINC01128 attenuated the progression of CRC by targeting and negatively regulating miR-363-3p, which provides a new reference for the prognosis of CRC.

## Supplementary Information

Below is the link to the electronic supplementary material.

Fig. 1 Supplementary. Prediction and analysis of LINC01128 downstream targets. A. Venn diagram of the predicted downstream targets of LINC01128. B-F. Expression of downstream targets of LINC01128 in normal and CRC samples. ^ns^P > 0.05, ***P < 0.001 (TIF 360 KB)

## Data Availability

Some or all data used during the study are available from the corresponding author by request.
