# Supplementary figures and images for: Upregulated lncRNA LINC01128 in colorectal cancer accelerates cell growth and predicts malignant prognosis through sponging miR-363-3p
Source: J Cancer Res Clin Oncol. 2024 May 26;150(5):276. doi: 10.1007/s00432-024-05804-4 (PMC11128396; doi:10.1007/s00432-024-05804-4)

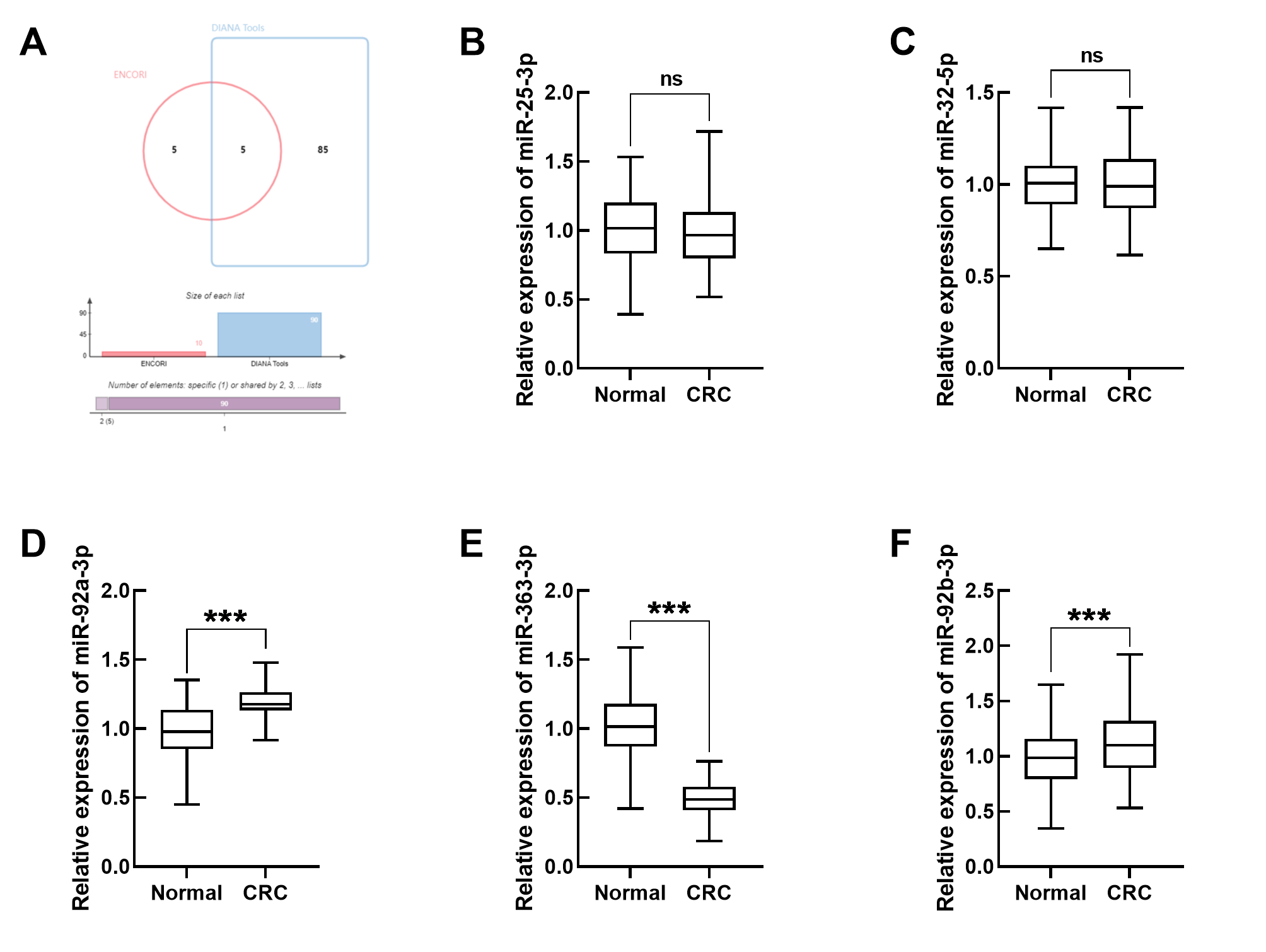

Supplement: Supplementary file 1 — Fig. 1 Supplementary. Prediction and analysis of LINC01128 downstream targets. A. Venn diagram of the predicted downstream targets of LINC01128. B-F. Expression of downstream targets of LINC01128 in normal and CRC samples. nsP > 0.05, ***P < 0.001 (TIF 360 KB) [file 432_2024_5804_MOESM1_ESM.tif]
